# Improving Food-Related Quality of Life in Inflammatory Bowel Disease through a Novel Web Resource: A Feasibility Randomised Controlled Trial

**DOI:** 10.3390/nu14204292

**Published:** 2022-10-14

**Authors:** Selina R. Cox, Wladyslawa Czuber-Dochan, Catherine L. Wall, Hazel Clarke, Candice Drysdale, Miranda C. Lomer, James O. Lindsay, Kevin Whelan

**Affiliations:** 1 Department of Nutritional Sciences, King’s College London, London SE1 9NH, UK; 2 Midwifery and Palliative Care, Florence Nightingale Faculty of Nursing, King’s College London, London SE1 8WA, UK; 3 Departments of Gastroenterology and Dietetics, Guy’s & St Thomas NHS Foundation Trust, London SE1 7EH, UK; 4 Department of Gastroenterology, Royal London Hospital, Barts Health NHS Trust, London E1 1BB, UK

**Keywords:** inflammatory bowel disease, food-related quality of life, eHealth

## Abstract

Food-related quality of life (FR-QoL) is impaired in inflammatory bowel disease (IBD) and education and support on food-related issues in IBD is needed. This feasibility trial aimed to investigate the effectiveness and acceptability of a web resource in enhancing FR-QoL in newly diagnosed IBD. Patients diagnosed with Crohn’s disease or ulcerative colitis in the preceding 12 months, with an impaired FR-QoL, were recruited and randomised to either receive access to the web resource (covering IBD-specific diet concerns) or no access (control group) for 12 weeks, while receiving usual clinical care. FR-QoL, health-related quality of life, psychological outcomes, and clinical disease activity were assessed. Web resource usage was assessed, and patients’ experiences of the web resource were investigated in semi-structured interviews. Of 81 patients screened, 50 participants were randomised, 30 to the web resource and 20 to control. FR-QoL increased more in the web resource (+11.7 SD 18.2) than control group (+1.4 SD 20.4) (*p* = 0.067), while IBD distress reduced in the web resource (−6.8 SD 26.6) and increased in the control group (+8.3 SD 25.5) (*p* = 0.052), albeit not statistically significantly. End of trial Crohn’s disease clinical activity (PRO-2) was significantly lower in the web resource than control group (*p* = 0.046). Participants most frequently accessed web resource content discussing dietary management of gut symptoms and in semi-structured interviews, reported the website to contain relevant information. This feasibility study demonstrates potential effectiveness of the web resource on improving FR-QoL and psychological outcomes in IBD. An adequately powered effectiveness RCT is feasible to conduct and is now warranted. NCT03884686.

## 1. Introduction

Dietary behaviours and intake are known to be impacted by inflammatory bowel disease (IBD), with a recent analysis showing around three quarters of patients restrict food and drink perceived to trigger gut symptoms [1]. Indeed, a recent systematic review of 29 studies reported 41–93% of patients engage in restrictive dietary behaviours [2]. Therefore, unsurprisingly, impaired nutrient intakes and suboptimal nutritional status are common in IBD [3,4], with a systematic review showing intakes of energy, fibre, folate and calcium are lower than recommended intakes, and fibre intakes are lower in IBD than healthy controls [5].

In addition to the important physiological roles of nutrients, food, diet, eating and drinking perform many social, emotional, and psychological roles in daily life [6], termed food-related quality of life (FR-QoL). IBD can result in disturbances in these psychosocial aspects of food and eating. Perceived food-related gut symptoms may cause anxiety regarding the likelihood of IBD relapse and resulting dietary restrictions may lead patients to avoid or feel excluded from social occasions and cultural traditions involving food [7]. This can limit the pleasure derived from food and eating and increase psychological stress [8,9,10]. Patients also described a lack of consistent information on diet in IBD and mixed experiences of access to dietary advice. This need for unambiguous dietary information is in line with a priority setting partnership of patients and clinicians, in which 250 diet-related uncertainties were raised by patients with IBD [11].

In order to manage this lack of information and support, some patients develop (mal)adaptive behaviours in relation to food and eating and drinking. For example, identifying suitable foods, checking food labels, preparing separate meals, fasting or avoiding social eating and ensuring proximity to toilets when eating outside the home [7].

Numerous studies have measured FR-QoL in IBD using a questionnaire specifically developed and validated in IBD [12]. The largest study to date in 1221 outpatients with IBD in the United Kingdom [13], reported a high prevalence of impaired FR-QoL in IBD, with lower scores associated with the number of recent disease flares, reduced IBD-specific quality of life and greater IBD-related distress, with impaired FR-QoL being associated with lower intakes of fibre and several micronutrients. Similar low FR-QoL scores have been observed in people with IBD in both the United States [14], Australia [15], New Zealand [16], and Turkey [17]. A study in active and inactive IBD [3] showed that the strongest predictor of FR-QoL was IBD symptom severity.

The impairment of FR-QoL in IBD, along with the patient-reported lack of clear and specific dietary information, indicates that resources and support specifically addressing this issue are warranted. This is especially important given that impaired FR-QoL occurs even during periods of disease remission and may compromise nutrient intakes [13].

A process of experience-based co-design (EBCD) was used to identify and develop approaches to supporting people with IBD with impaired FR-QoL, based upon which a web resource was developed [18]. The use of ‘eHealth’ technologies, including web or app-based remote patient treatment and monitoring (so-called ‘telemedicine’) and web-based patient resources, is increasing in IBD [19]. eHealth may encourage a participatory role of patients in their condition, facilitating greater patient control, which in turn may improve symptoms, psychological well-being, and healthcare utilisation [20].

eHealth interventions should be developed in conjunction with the target population and its development, refinement, and optimisation, including useability, should be explored following which the effectiveness should be formally investigated [20]. Such an approach has recently been utilised to show preliminary feasibility and acceptability of a web-based decision aid in encouraging patient involvement in ulcerative colitis (UC) management [21].

Since the web resource to address FR-QoL in IBD is entirely novel, a study was required to investigate the feasibility of a future effectiveness RCT, particularly with regard to patient willingness to participate, recruitment rates, and administering the intervention, and to explore the acceptability of the intervention and scope for optimisation [22,23].

The aim of this study was therefore to conduct a feasibility RCT of the novel FR-QoL web resource in patients with newly diagnosed IBD, and specifically to: (i) investigate the useability, acceptability, and feasibility of the website; and (ii) investigate the preliminary effectiveness of the website in improving impaired FR-QoL to inform sample sizes for a future RCT.

## 2. Materials and Methods

This was a randomised controlled feasibility study of a novel web resource to address FR-QoL in patients with newly diagnosed Crohn’s disease (CD) and UC.

### 2.1. Web Resource (Intervention)

The web resource was developed using an adapted EBCD approach [18]. This utilised qualitative data from individual interviews conducted with IBD patients (*n* = 15) and two focus group interviews with 11 healthcare professionals, consisting of four Consultant Gastroenterologists (Attending Gastroenterologists), two IBD specialist registrars (IBD Fellows), two IBD specialist nurses, two gastroenterology specialist dietitians, and one IBD psychologist. The main food-related issues in IBD requiring the most urgent attention were those around the need for information on food suitability and food triggers of gut symptoms, early FR-QoL-focused interventions for patients and their families, and tailored advice on individuals’ diet-related problems. Both patients and clinicians identified the need for reliable educational material regarding food-related issues, and that these should be from trusted sources, including both health professionals and patients who had overcome problems of FR-QoL and were therefore experts by experience.

The website was developed to consist of five sections containing fact sheets (9), links to external content (4 links including to relevant British Dietetic Association and Crohn’s and Colitis UK webpages) and short clinician videos (45 videos from gastroenterologists, IBD nurses, specialist dietitians) and patient videos (19 videos) embedded within the website. These resources consisted of both education and support on topics including symptoms of IBD, special diets, fibre, exclusive enteral nutrition, probiotics, managing specific gastrointestinal symptoms, and the impact of IBD on socialising and eating out. The website was fully accessible on any computer with internet access and on any mobile Smartphone (although not optimized for use for use on the latter).

### 2.2. Study Design and Participants

Patients with recently diagnosed IBD and impaired FR-QoL were recruited from two large gastroenterology clinics in London (Guy’s and St Thomas’ NHS Foundation Trust and Barts Health NHS Trust), United Kingdom.

Patients were eligible if they were over 16 years of age and were recently (within previous 12 months) diagnosed with CD or UC via endoscopy, were not consuming a medically prescribed diet, and had access to and ability to use the internet on a computer. Patients were excluded if they had a diagnosis of indeterminate colitis, were hospitalised or in any form of institutionalised living, were receiving enteral nutrition supplying more than 50% of energy requirements, had significant co-morbidities likely to influence dietary intake (e.g., diabetes or coeliac disease), or were pregnant or less than 6 months postpartum. To recruit patients with food-related issues relating to their IBD and who may benefit from intervention, FR-QoL was assessed as part of screening using the validated FR-QoL-29 questionnaire [12]. Patients with a score of >90 (out of a possible 145), representing impaired FR-QoL, were excluded.

Research ethics committee approval was received from the East Midlands–Nottingham 1 Research Ethics Committee (Reference 18/EM/0307) on 9 November 2018. The trial was registered on ClinicalTrials.gov (identifier NCT03884686).

### 2.3. Randomisation

Consenting participants were randomised to continue receiving all currently available dietary support from their team (‘usual care’ control group) or to additionally receive access to the web resource (‘web resource’ intervention group). Randomisation was stratified by disease (CD or UC) and recruitment site (Guy’s and St Thomas’ NHS Foundation Trust or Barts Health NHS Trust) and the list was produced by a researcher not involved in recruitment using a random allocation generator with a 1:1 ratio (randomisation.com; accessed on 1 December 2018). Individual allocation was sealed into opaque envelopes and opened once all baseline data collected. Due to the nature of the intervention and control treatments, the participants and researchers could not be blinded to allocation.

### 2.4. Trial Protocol and Procedures

A key objective was to investigate the optimal recruitment approaches for this trial, and therefore a variety of techniques were adopted and their effectiveness at resulting in participant recruitment were recorded. Approaches consisted of: (i) researchers attending gastroenterology clinics without prior screening of lists to identify potentially eligible patients (‘untargeted clinic recruitment’); (ii) researchers screening gastroenterology clinic lists remotely and then attending only those clinics with potentially eligible patients (‘targeted clinic recruitment’); (iii) clinicians (gastroenterologists, IBD nurses, dietitians, pharmacists) identifying potentially eligible patients during routine clinical appointments (‘clinician referral’); (iv) sending a letter from the gastroenterology department inviting newly diagnosed patients to be screened, with a reminder letter 6–8 weeks later to non-responders (‘letter and self-referral’); and (v) advertising and inviting patients to contact researchers via the Crohn’s and Colitis UK ‘Take Part In Research’ webpage (‘advertising and self-referral’).

Eligible patients provided written informed consent before the collection of demographic and clinical information. Patients then completed baseline questionnaires either in person or online (QualtricsXM 2019). After completion of the questionnaires, patients were randomised to one of the two study groups. Patients in the web resource group received a link to the web resource, with a unique username and password, and were encouraged to access it regularly for 12 weeks, in addition to all usual support from their clinical team. Patients in the control group (usual care only) were instructed to continue to access all usual support from their clinical team for 12 weeks.

After 12 weeks, patients in both groups were emailed a link to complete end of trial questionnaires online (QualtricsXM 2019). Patients were given three days to complete the questionnaires, before a reminder email was sent. Following completion of the questionnaires, patients in the control group were given a unique username and password to access the website for 4 weeks.

Patients in the web resource group were invited to attend a 45–60 min semi-structured interview regarding acceptability of the web resource, the trial, and perceived impact on FR-QoL.

### 2.5. FR-QoL, HR-QoL and Psychological Outcomes

Several questionnaires were administered at baseline and end of trial to assess preliminary effectiveness of the web resource. FR-QoL was measured using the validated FR-QoL-29 questionnaire [12]. Disease-specific health-related quality of life (HR-QoL) was measured using the UK IBD-Q [24] and the level of distress experienced in relation to IBD-specific problems was measured using the IBD Distress Scale (IBD-DS) [25], because poorer scores on these outcomes have been associated with lower FR-QoL [13]. Anxiety and depression were measured using the hospital anxiety and depression scale (HADS) [26]. End of trial scores and change in scores during the 12-week trial were compared between intervention and control groups.

### 2.6. Disease Activity and Disease Control

In patients with CD, disease activity was assessed at baseline and end of trial using the patient-reported outcome measure 2 (PRO-2) [27]. This adaptation of the Crohn’s Disease Activity Index is calculated from abdominal pain severity and liquid or very soft stool frequency in a 7-day period and has been shown to correlate with CDAI scores [27]. In patients with UC, disease activity was assessed using the Partial Mayo score [28], which assesses the non-invasive elements of the Mayo Score: stool frequency, rectal bleeding and physician global assessment of disease activity. Patient-perceived control of IBD was measured using the IBD-control questionnaire at baseline and end of trial [29].

### 2.7. Acceptability and Usage of the Web Resource

An additional consent form was signed by participants from the intervention group agreeing to take part in the semi-structured interviews after the trial regarding acceptability of the web resource. Interviews were conducted by two researchers using a standardised topic guide, focusing on the patient’s use of the website during the trial, the perceived effectiveness of the website on FR-QoL, their opinions on the website content and suggestions for website improvement. Interviews were transcribed verbatim using the dictate function within Microsoft Word and were thoroughly checked and corrected by a researcher (not involved in conducting interviews) [30]. The interviews were then analysed using ideographic interpretative phenomenological analysis [31]. An experienced qualitative researcher read the transcripts twice and noted the striking issues or topics emerging. The themes were then named, based upon the perceived meaning of the responses and how these compared with other responses within the interview. Overarching, or ‘super-ordinate’ themes were identified, and the themes of all interviews were amalgamated.

Each participant was assigned a unique website username and password, enabling analysis of website usage for each participant using Google Analytics. Data on the frequency and duration of usage were collected at three different levels: the whole website, individual sections, and individual written resources, videos, and links to external websites. A video view was recorded once ≥25% of the video had been watched, with shorter viewing not considered a sufficiently meaningful engagement.

### 2.8. Patient Identification, Screening, Randomisation and Completion

For each patient screened, data was collected on identification method (untargeted clinic recruitment, targeted clinic recruitment, clinician referral, letter and self-referral, advertising and self-referral), hospital site, reasons for exclusion, willingness to participate, and the number of randomised participants completing the trial and completing each questionnaire. 

### 2.9. Statistical Analysis

This feasibility RCT aimed to generate data upon which to perform a sample size calculation for a full-scale effectiveness RCT, therefore a power-based sample size calculation was not performed here [32]. Based on the rule of thumb that at least 12 participants per group is sufficient for a pilot study [33], along with the findings of similar feasibility studies, a sample of 50 patients (25 per arm) was deemed sufficiently large to provide useful information about the feasibility aspects of the study, while allowing for attrition [34].

Demographic and IBD information were compared between groups (intervention vs. control) at baseline and presented as mean (standard deviations, SD) for continuous variables and *n* (%) for categorical variables. The primary outcome measure (FR-QoL-29 score) and secondary outcome measures (scores for UK IBD-Q, IBD-DS, HADS, PRO-2, Partial Mayo Score, IBD-control) were calculated as both absolute values and change from baseline values and compared between groups at end of trial using an unpaired *t*-test or Mann–Whitney test for normally and non-normally distributed continuous data. Although as yet there is no minimal clinically important difference for the FR-QoL-29, the proportion of patients achieving: (1) score of >90 points; and (2) an increase in ≥10 points at end of trial were calculated as a gauge of clinical significance. For participants withdrawing from the study prematurely, data were analysed intention to treat (ITT) by carrying forward available baseline data.

Feasibility data (such as the number of patients screened and the proportion consenting to and completing the trial) are presented as number of patients and percentages. The data were analysed using IBM SPSS version 26. Differences between groups were considered statistically significant when *p* ≤ 0.05.

## 3. Results

**Figure 1:**
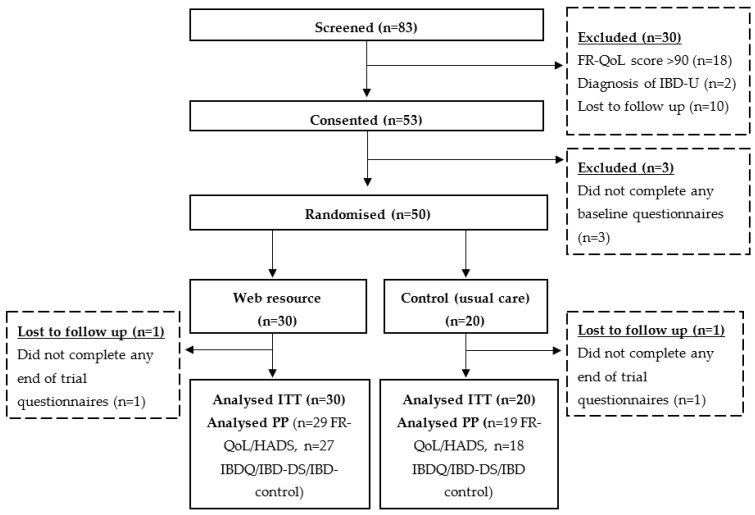
CONSORT diagram showing patient flow during the trial.

Participant recruitment took place between December 2018 and February 2020. During this time, 83 patients underwent full screening, and fifty patients were randomised, 30 to the intervention group and 20 to the control group (Figure 1). Block randomisation was not used, thus explaining the unequal group allocation. Two participants failed to complete any end of trial questionnaires (one in the intervention group, one in control group), therefore no end of trial data is available for these participants. Three participants (two in the intervention group, one in the control group) completed FR-QoL and HADS, but not the remaining end of trial questionnaires. The ITT analysis therefore consists of 50 patients overall (30 intervention, 20 control) with baseline data carried forward. The per protocol (PP) analysis therefore includes 48 patients (29 intervention, 19 control) for FR-QoL-29 and HADS, and 45 patients (27 intervention, 18 control) for the UK IBD-Q, IBD-DS, IBD-control and PRO-2/Partial Mayo.

There were no differences in demographic or clinical characteristics between study groups at baseline, except for a greater proportion living in rented accommodation in the control group (15/20, 75%) compared to the intervention group (11/30, 37%) (*p* = 0.029) and there were more current smokers in the control group (7/20, 35%) compared to the intervention group (1/30, 3%) (*p* = 0.008) (Table 1 and Table 2).

### 3.1. Food-Related Quality of Life (FR-QoL)

**Figure 2:**
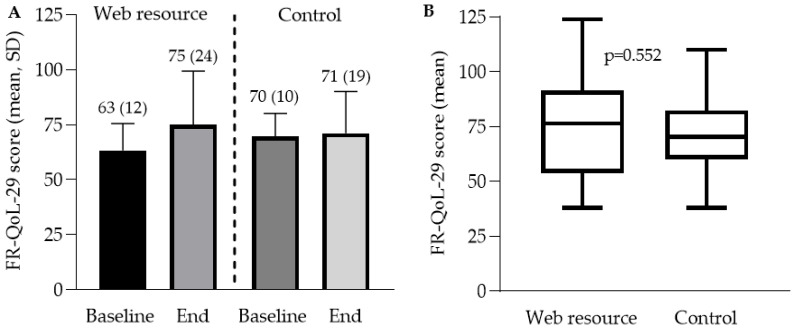
(**A**) Baseline and end of trial FR-QoL-29 scores; (**B**) End of trial FR-QoL-29 scores.

In the ITT analysis, there was no difference in end of trial FR-QoL-29 score in the intervention (75.0, SD 24.3) compared with the control group (71.1, SD 19.0, *p* = 0.552) (Table 3, Figure 2B). There was a greater increase in FR-QoL-29 score during the trial in the intervention (+11.7, SD 18.2) compared with the control group (+1.4, SD 20.4), but this did not reach statistical significance (*p* = 0.067). There was no statistical difference in the proportion of patients achieving an FR-QoL-29 score >90 at end of trial between the intervention (7/30, 23%) and control (3/20, 15%) (*p* = 0.720) nor in the proportion achieving a ≥10-point increase in total score between intervention (15/30, 50%) and control (6/20, 30%) (*p* = 0.160).

### 3.2. Quality of Life and Psychological Outcomes

The ITT analysis revealed no significant differences between intervention and control groups in end of trial IBD-Q (77.5, SD 13.1 vs. 78.4, SD 11.3, *p* = 0.821) or HADS scores (13.4, SD 7.9 vs. 12.9, SD 4.7, *p* = 0.779), or change in scores during the trial for IBD-Q (+7.6, SD 10.0 vs. +9.8, SD 19.3, *p* = 0.607) or HADS (−3.8, SD 5.7 vs. −3.8, SD 7.9, *p* = 0.986) (Table 3). 

There was no significant difference in end of trial IBD distress score between groups (91.1, SD 35.4 vs. 94.5, SD 29.9, *p* = 0.727). However, there was a greater reduction in IBD distress in the intervention compared with the control group in both the ITT (−6.8, SD 26.6 vs. +8.3, SD 25.5, *p* = 0.052) and PP (−7.5, SD 28.0 vs. +9.2, SD 26.7, *p* = 0.053) analysis, respectively, although neither reached statistical significance.

### 3.3. Disease Activity and Disease Control

In the ITT analysis, there were no significant differences in end of trial IBD-control score in intervention (73.2, SD 33.2) compared with control group (69.7, SD 24.5) (*p* = 0.727) or change in score during the trial (+8.2, SD 26.6 vs. +5.6, SD 34.4, *p* = 0.761) (Table 3).

In the ITT analysis, end of trial PRO-2 score (CD only) was significantly lower in the intervention group (3.1, SD 2.8) compared with the control group (5.2, SD 1.3) (*p* = 0.046). However, there was no significant difference between groups in the change in PRO-2 score during the trial (+0.2, SD 3.6 vs. +1.2, SD 3.1, *p* = 0.607).

In the ITT analysis, there were no significant differences in end of trial Partial Mayo score (UC only) in intervention (1.3, SD 1.3) compared with the control group (1.0, SD 1.0) (*p* = 0.475) or change in score during the trial (−0.4, SD 1.3 vs. −0.9, SD 1.8, *p* = 0.301) (Table 3).

### 3.4. Acceptability and Usage of the Web Resource

Six participants from the intervention group agreed to take part in semi-structured qualitative interviews, which were conducted face-to-face (*n* = 4) or over the telephone (*n* = 2). Median age of interviewed participants was 32 (IQR 11.5 years), 5/6 (83%) were women, and 2/6 (29%) had CD. In total, 206 min of data were collected (mean interview duration 34:37 min, range 18:11–45:39 min). The themes emerging from the interviews were: ‘Content of the intervention’, ‘Format, structure and navigation’, ‘Suggestions for change’. Verbatim quotes are used to better illustrate the themes.

Participants commented favourably on website content, particularly regarding IBD, different diets, healthy eating, fibre, vitamin and mineral supplementation, and socialising and going out. As newly diagnosed patients, information on socialising and eating out was particularly helpful in reducing anxiety and stress related to these occasions.

Participants found the format and structure of the website very clear, easy to access and navigate. They liked the depth and different formats of the information, and the fact it was presented by both clinicians and patients: “*It was really easy to use and easy to navigate. I really liked it. I loved the videos; the videos are great*”. (F, UC, 34). Most participants accessed the intervention once or twice a week, or more often at the beginning of the intervention. Some tended to get through the website content in one session, while others preferred to access content in short sessions, e.g., during lunch breaks and commutes.

Participants made several suggestions to improve the website. Some wanted more patients’ videos, while others suggested including more factsheets that could be downloaded. One participant suggested adding a brief synopsis of the videos, and one suggested making the information for people with CD and UC more balanced: “*I just zone out if I see Crohn’s (information) now. (…) It’s like ‘it’s not me’. I’ll ignore that. So, it would obviously benefit me if it did focus more on UC or at least equal if it can*.” (F, UC, 30)

Data on the usage of the website was available for 25 of the 30 patients randomised to the web resource group. Five patients either did not access the website during the intervention or Google Analytics had been blocked by their web browser. A further six patients logged into the website but did not access any content (i.e., did not watch any videos, or access any information pages).

Among patients who logged into the website at least once during the trial, the number of website logins was mean 4.2 (SD 2.9) and the total time spent on the website during the trial was 27 min, 51 s (SD 21 min, 15 s), while the average time spent on the website per visit was 13 min, 41 s (SD 15 min, 1 s). The number of patients accessing each section of the website is presented in Table 4.

The section with content accessed by the greatest number of patients covered the dietary management of specific gut symptoms (13/25, 52%). The most frequently accessed sections were those covering disease symptoms and the impact of IBD on family, socialising and eating out, while the least frequently accessed section was that containing information on special dietary approaches in IBD, covering subjects such as exclusive enteral nutrition, probiotics, special diets, and micronutrients.

The most frequently played patient videos were regarding the importance of dietary fibre in IBD, whether IBD activity can be altered through diet and experience of consuming alcohol in IBD. Of the four links to external information, the most frequently accessed was the Crohn’s & Colitis UK webpages on bloating and wind and on diarrhoea and constipation.

### 3.5. Patient Identification, Screening, Randomisation and Completion

Of the 83 participants screened, 18 (22%) were excluded due to an FR-QoL score >90 and 2 (2%) had IBD-U. Of the 63 people who were otherwise eligible, 10/63 (16%) failed to return consent forms despite reminders, and 3/63 (5% of consented) did return consent forms but then failed to complete baseline questionnaires and were not randomised.

The target sample size of 50 participants randomised was achieved (Figure 1). Two participants (one from each study group) failed to return any end of trial questionnaires representing a complete attrition of only 4%. Furthermore, three participants (2/30 (7%) in intervention, 1/50 (5%) in control group), completed only some of the end of trial questionnaires. Therefore, end of trial data was missing or incomplete for a total of 5/50 (10%) patients. Numbers of patients screened and recruited through each method at each recruitment centre, success of end of trial data completion, and overall monthly recruitment rate, are presented in Table 5.

Across both sites, the most successful method of patient identification and recruitment was ‘untargeted clinic recruitment’ (26/50 (52%) patients) followed by targeted clinic recruitment (21/50 (42%)). In contrast, only nine patients self-referred after receiving an invitation letter, three of whom (33%) were randomised (Table 5).

## 4. Discussion

The concept of FR-QoL is gaining increasing recognition in IBD, in which food is associated with gut symptoms which result in dietary restrictions and difficulties in enjoying food and the extensive range of occasions revolving around it [2]. This feasibility RCT represents the first attempt at improving impaired FR-QoL known to be prevalent in IBD [13,14,15], using an intervention that was co-developed with patients and professionals. Despite this being a feasibility study in only 50 patients, use of the website resulted in a non-significant increase in FR-QoL and reduction in IBD distress, indicating the potential to improve FR-QoL and concurrently reduce psychological distress. Our study demonstrates that the intervention is acceptable to patients with IBD and participant recruitment and retention to such a trial are feasible. Together, the preliminary effectiveness, acceptability, usability, and feasibility findings indicate that a fully powered RCT is warranted.

Although the study was not powered to detect differences in effectiveness, the intervention showed promising findings for FR-QoL and IBD distress. A greater, albeit non-significant (*p* = 0.067), increase in FR-QoL-29 score in the web resource group (+11.7 points) compared with the control group (+1.4 points) signals a positive impact of the intervention on FR-QoL and suggests the need for an appropriately powered RCT to confirm these effects. Only patients with an FR-QoL-29 score of ≤90 were enrolled, meaning the effects of the web resource in patients with only slightly impaired FR-QoL score (>90) is currently unknown. However, in a recent large survey in IBD, the average FR-QoL score was 80.8 [13], and therefore the inclusion of patients with a FR-QoL ≤90 is likely to capture a large proportion of patients.

Patients with active Crohn’s disease have been shown to score higher on measures of disordered eating than the general population [35]. Furthermore, 10.2% of patients with IBD were previously found to meet the criteria for avoidant/restrictive food intake disorder (ARFID), although clinician recognition of this was low [36]. Some items on disordered eating scales (e.g., Binge Eating Scale, Control of Eating Questionnaire, and Nine-Item Avoidant/Restrictive Food Intake Disorder Screen) overlap with only a minority of items on the FR-QoL-29 (e.g., avoidance of foods due to perceived risk of GI symptoms), representing the differences in these phenomena. However, there are many differences in the reasons for and prevalence of disordered eating compared with poor FR-QoL. Clinicians treating IBD should be trained in the indicators of disordered eating compared with impaired FR-QoL in order to manage either appropriately.

Since this study aimed to investigate a novel intervention and represented the first attempt at improving impaired FR-QoL in IBD, an important outcome was the feasibility and acceptability of recruiting participants and administering the web resource. The recruitment target of 50 patients was achieved, and a relatively low attrition rate of 4% was observed. A small number of patients failed to complete end of trial questionnaires despite multiple reminders, though this is perhaps unsurprising given the use of remote electronic questionnaires. An assessment of patients with asthma found that key enablers to completing an electronic health questionnaire included ease and convenience of completion, patient-perceived priority and usefulness of the questionnaire and its findings [37].

Patients accessed website content an average of 4.2 times during the 12-week trial. The most frequently accessed webpage covered the dietary management of gut symptoms, including bloating and flatulence, abdominal pain and diarrhoea and constipation. The most frequently viewed diet-related videos were those concerning dietary fibre, altering IBD activity through diet and consuming alcohol. These findings corroborate the semi-structured qualitative interviews in which these topics were highlighted by participants as particularly pertinent. The interest in the management of gut symptoms through diet is unsurprising given that this is a known area of concern and priority for patients [11]. In the largest survey of FR-QoL in IBD to date, nearly three quarters of respondents had impaired FR-QoL related to the effects of eating on gut symptoms, with a detrimental effect on enjoyment of food [13]. Overall, participants found the website to include relevant and useful information at an appropriate depth, which was easy to access and navigate.

Patients with IBD have previously voiced concerns regarding reliable sources of dietary information for IBD [11,38] and a systematic review demonstrated restrictive dietary behaviours in IBD to be associated with dietary misinformation, among other factors [2]. In a qualitative analysis, patients with IBD revealed that they would benefit from IBD educational videos and access to forums in which patients could share their experiences [39]. However, conflicting, overwhelming, and unreliable information accessed via the internet caused anxiety in some patients. The use of alternative health websites and other non-official websites found through a search engine has been associated with greater anxiety in IBD [40]. Therefore, this novel, evidence-informed, patient and professional co-developed website may be of great potential in IBD.

The most successful methods of recruitment were attending gastroenterology clinics, with ‘targeted clinic recruitment’ appearing most effective in identifying suitable patients. ‘Targeted clinic recruitment’ involved pre-screening clinic lists to identify patients most likely to be eligible, therefore reducing the number of unsuccessful screening attempts and unnecessary clinic attendance, and therefore being more time efficient. Recruiting through ‘invitation letters and self-referral’, ‘clinician referral’ and ‘advertising and self-referral’, were considerably less successful, although were less time intensive. A review of qualitative studies revealed that organisational difficulties and scarcity of clinician time, as well as issues relating to equipoise and patient eligibility, impeded trial recruitment rates [41]. A systematic review of 61 clinical trials has demonstrated online recruitment to be more effective in terms of recruitment rate, costs, and time efficiency, however in-person clinic recruitment (offline) was superior in terms of conversion rates [42]. In a future full-scale RCT of the web-based FR-QoL resource, a combination of online and in-person recruitment methods should be considered.

### 4.1. Future Research

This feasibility RCT suggests that a full-scale RCT of a web resource for FR-QoL is warranted in IBD. The target audience for the website requires consideration however. In the interest of defining a specific study population, we included only patients diagnosed with IBD in the preceding 12 months, however a future RCT should aim to include patients with a longer diagnosis, many of whom continue to experience challenges with FR-QoL. A recent survey in IBD demonstrated impaired FR-QoL, despite an average duration of diagnosis of 12.5 years, indicating that an FR-QoL intervention would be relevant in patients with a longer-standing diagnosis [13]. Furthermore, only 32% of patients had active disease at the time of the survey, and although currently active disease was negatively associated with FR-QoL, average FR-QoL score was below 90 even in patients in remission. Disease activity was not assessed by objective measures such as faecal calprotectin or endoscopy, therefore some of the patients with active disease as measured by symptom-based indices in this study may have had functional-like GI symptoms. A full-scale RCT of the web resource should aim to perform subgroup analyses of the effectiveness in active and inactive disease, assessed through objective markers, and between CD and UC, where diverse food-related issues may exist. Although patients with a history of GI resections, current stomas, and short bowel syndrome were included in this study, the numbers were insufficient to perform subgroup analyses. Unique dietary recommendations exist in these circumstances, so a future RCT should aim to assess FR-QoL and the effectiveness of the website in these clinically diverse patients. Finally, some patients expressed during both the development of the intervention and in the interviews following the intervention in this study that little importance was attributed to diet by clinicians treating their IBD. Future research should therefore aim to develop evidence-based resources regarding FR-QoL aimed at clinicians and healthcare professionals treating IBD, in addition to patient-facing resources.

### 4.2. Study Limitations

Some limitations of this feasibility study should be noted. While the chosen sample size is relatively large for a feasibility study [33], some patients failed to complete end of trial questionnaires, thus reducing statistical power in these outcomes, which of course was not the aim in the current study. Furthermore, as discussed above, the website was designed specifically for patients with IBD diagnosed in the preceding 12 months and should be expanded to investigate the potential effectiveness in patients with longer IBD diagnoses in a future RCT. The lack of objective markers of disease activity raises challenges in accurately defining active and inactive IBD. Finally, although the aim of website is not to replace or mimic dietetic intervention, prior access to dietetic services could be a confounding factor. This information was not collected here but a future RCT should gather this information.

This is the first trial to investigate the effectiveness of an intervention, developed through experience-based co-design, on impaired FR-QoL in IBD. This has shown that a full-scale RCT of a web resource for FR-QoL is feasible and warranted in IBD, and semi-structured qualitative interviews provide valuable patient acceptability data that can be used to optimise the intervention for such a future RCT.

## Figures and Tables

**Table 1:** Demographic characteristics of the study groups.

	Web Resource (*n* = 30)	Control (*n* = 20)	*p*-Value
Age (years), mean (SD)	34 (11)	30 (6)	0.141
Male	14 (47)	12 (60)	0.355
**Ethnicity**			0.705
Asian or Asian British	6 (20)	4 (20)	
Black, Black British, Caribbean or African	1 (3)	0 (0)	
Mixed or multiple ethnic groups	0 (0)	0 (0)	
White	22 (73)	16 (80)	
Other ethnic group	1 (3)	0 (0)	
**Education level**			0.159
No formal qualifications	1 (3)	4 (20)	
School level qualifications	3 (10)	1 (5)	
Advanced school level qualifications	3 (10)	0 (0)	
University Degree	17 (57)	13 (65)	
Postgraduate Degree	6 (20)	2 (10)	
**Relationship status**			0.856
Married	12 (40)	8 (40)	
Living with partner	8 (27)	5 (25)	
Divorced	1 (3)	0 (0)	
Single	9 (30)	7 (35)	
**Accommodation status**			**0.029**
Homeowner	12 (40) ^a^	3 (15) ^a^	
Renting	11 (37) ^a^	15 (75) ^b^	
Living with family	7 (23) ^a^	2 (10) ^a^	
**Employment status**			0.850
Full-time employed	20 (67)	16 (80)	
Part-time employed	1 (3)	1 (5)	
Full-time education	2 (7)	1 (5)	
Home duties	2 (7)	1 (5)	
Retired	1 (3)	0 (0)	
Unemployed	4 (13)	1 (5)	
**Smoking status**			**0.008**
Current smoker	1 (3) ^a^	7 (35) ^b^	
Previous smoker	11 (37) ^a^	3 (15) ^a^	
Non-smoker	18 (60) ^a^	10 (50) ^a^	

All data are *n* (% of group) unless otherwise stated. Groups were compared using chi-squared test (categorical) or unpaired *t*-tests (continuous). Columns without superscripts in common are significantly different (*p* < 0.05).

**Table 2:** Clinical characteristics of the study groups.

	Web Resource (*n* = 30)	Control (*n* = 20)	*p*-Value
**Diagnosis**			0.186
Crohn’s disease	13 (43)	5 (25)	
Ulcerative colitis	17 (57)	15 (75)	
**Crohn’s disease location**			0.990
Ileal (L1)	5 (17)	2 (10)	
Colonic (L2)	3 (10)	1 (5)	
Ileocolonic (L3)	5 (17)	2 (10)	
**Crohn’s disease behaviour**			0.758
Non-stricturing, non-penetrating (B1)	9 (69)	3 (60)	
Stricturing (B2)	3 (23)	1 (20)	
Penetrating (B3)	1 (8)	1 (20)	
Baseline PRO-2 score, mean (SD)	2.9 (2.8)	4.0 (3.3)	0.469
**Ulcerative colitis extent**			0.328
Proctitis (E1)	7 (41)	3 (20)	
Left-sided (E2)	4 (24)	3 (20)	
Extensive (E3)	6 (35)	9 (60)	
**Ulcerative colitis severity**			0.279
Clinical remission (S0)	9 (53)	4 (27)	
Mild (S1)	6 (35)	7 (47)	
Moderate (S2)	2 (12)	4 (27)	
Baseline Partial Mayo Score, mean (SD)	1.7 (1.6)	2.2 (1.9)	0.405
**Age of IBD onset**			0.214
17–40 years	25 (83)	19 (95)	
>40 years	5 (17)	1 (5)	
IBD diagnosis (years), mean (SD)	0.4 (0.2)	0.5 (0.3)	0.548
Previous surgery	2 (7)	0 (0)	0.510
**Current medications at randomisation**			
5-aminosalicylates	12 (40)	5 (25)	0.365
Thiopurines	7 (23)	3 (15)	0.720
Methotrexate	1 (3)	0 (0)	1.000
Biologics	8 (27)	3 (15)	0.489
Corticosteroids	4 (13)	3 (15)	1.000
Allopurinol	1 (3)	1 (5)	1.000
Baseline FR-QoL-29 score, mean (SD)	63.2 (12.3)	69.7 (10.5)	0.060

All data are *n* (% of group) unless otherwise stated. Groups were compared using chi-squared test (categorical) or unpaired *t*-tests (continuous).

**Table 3:** Food-related quality of life, health-related quality of life, psychological and clinical outcomes.

Questionnaire	Score at End of Trial			Change in Score from Baseline to End		
	Intervention	Control	*p*-Value	Intervention	Control	*p*-Value
Intention to Treat	(*n* = 30)	(*n* = 20)		(*n* = 30)	(*n* = 20)	
FR-QoL (FR-QoL-29)	75.0 (24.3)	71.1 (19.0)	0.552	+11.7 (18.2)	+1.4 (20.4)	0.067
IBD HRQoL (IBD-Q)	77.5 (13.1)	78.4 (11.3)	0.821	+7.6 (10.0)	+9.8 (19.3)	0.607
IBD Distress (IBD-DS)	91.1 (35.4)	94.5 (29.9)	0.727	−6.8 (26.6)	+8.3 (25.5)	0.052
HADS	13.4 (7.9)	12.9 (4.7)	0.779	−3.8 (5.7)	−3.8 (7.9)	0.986
IBD-Control	73.2 (33.2)	69.7 (24.5)	0.559	+8.2 (26.6)	+5.6 (34.4)	0.761
CD activity (PRO-2)	3.1 (2.8)	5.2 (1.3)	**0.046**	+0.2 (3.6)	+1.2 (3.1)	0.607
UC activity (Partial Mayo)	1.3 (1.3)	1.0 (1.0)	0.475	−0.4 (1.3)	−0.9 (1.8)	0.301
**Per protocol**						
FR-QoL (FR-QoL-29)	75.2 (24.7)	71.6 (19.4)	0.589	+12.1 (18.4)	+1.5 (21.0)	0.069
IBD HRQoL (IBD-Q)	77.0 (13.2), [*n* = 27]	76.9 (10.7) [*n* = 18]	0.969	+8.4 (10.2) [*n* = 27]	+10.8 (20.0) [*n* = 18]	0.601
IBD Distress (IBD-DS)	91.9 (36.8), [*n* = 27]	99.8 (26.3) [*n* = 18]	0.435	−7.5 (28.0) [*n* = 27]	+9.2 (26.7) [*n* = 18]	0.053
HADS	13.1 (7.8), [*n* = 29]	12.8 (4.8) [*n* = 19]	0.864	−4.0 (5.8) [*n* = 29]	−4.0 (8.1) [*n* = 19]	0.986
IBD-Control	71.6 (33.2)	69.5 (25.2)	0.819	+9.1 (27.9)	+6.2 (36.4)	0.760
CD activity (PRO-2)	3.1 (2.8)	5.5 (1.3)	**0.034**	+0.1 (2.5)	+0.3 (1.6)	0.738
UC activity (Partial Mayo)	1.4 (1.3)	1.1 (1.0)	0.431	−0.2 (1.0)	−0.8 (1.7)	0.224

Groups were compared using independent *t*-tests and *p*-values in bold are <0.05.

**Table 4:** Web resource usage data.

Topics	Number of Patients Accessing (% of Total) (*n* = 25)
1.1 What is IBD?	7 (28)
2.1 An introduction to diet and IBD	3 (12)
2.2 What is a healthy diet in IBD?	5 (20)
2.3 Can altering my diet help reduce IBD activity?	6 (24)
2.4 Eating during an IBD flare	5 (20)
2.5 Should I exclude foods from my diet?	8 (32)
2.6 Can I eat fruits and vegetables?	5 (20)
2.7 What types of fibre should I eat?	5 (20)
3.1 How can a liquid diet help with Crohn’s disease?	4 (16)
3.2 Practical advice for following a liquid diet	0 (0)
3.3 Should I take probiotics?	3 (12)
3.4 Should I follow a specific diet?	2 (8)
3.5 Why are iron, calcium and minerals important in IBD?	2 (8)
3.6 Which foods contain the vitamins and minerals I need?	3 (12)
4.1 Can changing my diet help manage symptoms? (overview)	6 (24)
4.2 Dietary management of gut symptoms	11 (44)
4.3 Identifying foods that trigger symptoms	6 (24)
5.1 Cooking for your family	5 (20)
5.2 Meal planning	4 (16)
5.3 Eating out and practical advice	5 (20)
5.4 Can I drink alcohol?	7 (28)
5.5 Guide for family and friends cooking for patients	4 (16)

**Table 5:** Recruitment rates according to recruitment method, overall and at the two recruitment centres.

	All Participants			Guy’s and St Thomas’ NHS Foundation Trust			Barts Health NHS Trust		
Recruitment Method	Screened	Randomised (% Screened)	All End of Trial Data Available *	Screened	Randomised (% Screened)	All End of Trial Data Available *	Screened	Randomised (% Screened)	All End of Trial Data Available *
Untargeted clinic recruitment	40	26 (65%)	24/26 (92%)	12	6 (50%)	6/6 (100%)	28	20 (71%)	18/20 (90%)
Targeted clinic recruitment	33	21 (64%)	18/21 (86%)	24	16 (67%)	13/16 (81%)	9	5 (56%)	5/5 (100%)
Clinician referral	1	0 (0%)	-	1	0 (0%)	-	0	-	-
Letter and self-referral	9	3 (33%)	3/3 (100%)	9	3 (33%)	3/3 (100%)	0	-	-
Advertising and self-referral	0	0 (0)	0 (0)	0	0 (0)	0 (0)	0	0 (0)	0 (0)
TOTAL	83	50 (60%) 3.3 per month	45 (90%)	46	25 (54%) 1.7 per month	22/25 (88%)	37	25 (68%) 2.1 per month	23/25 (92%)

* Data are presented as *n* (%) recruited via each method and for those completing all six end of trial online questionnaires. A description of the recruitment approaches can be found in the methods section.

## Data Availability

The data are not publicly available due to ethical limitations.
